# Determination of Tobacco Specific Hemoglobin Adducts in Smoking Mothers and New Born Babies by Mass Spectrometry

**Published:** 2007-08-06

**Authors:** Steven R. Myers, Md. Yeakub Ali

**Affiliations:** Department of Pharmacology and Toxicology, University of Louisville School of Medicine, 500 South Preston St. Louisville, KY 40292

**Keywords:** Biomarkers, Exposure, Nitrosoamine-hemoglobin adducts, Tobacco, Smoking, GC/MS

## Abstract

Biological markers for assessment of exposure to a variety of environmental carcinogens has been widely applied in both basic as well as clinical research. Exposure to tobacco smoke presents an ideal environment with which to develop, characterize, and refine biological markers, especially of those carcinogens found in tobacco. In the present study, a sensitive gas chromatography/mass spectrometry (GC/MS) method was developed to measure nitrosamine- hemoglobin adducts (HPB-Hb (4-Hydroxy-3-pyridinyl-1-butanone) at trace levels in red blood cells of both African-American and Caucasian smoking and nonsmoking mothers and their infants. Gas chromatographic and mass spectrometric methods with chemical ionization (CI) of methane reagent gas in both positive and negative ion mode as well as electron ionization (EI) were studied to determine differences in sensitivity of detection among the various ionization methods. Detection limits using both positive and negative chemical ionization modes were found to be 30 femtomoles of HPB, whereas detection using electron impact modes yielded a detection limit of 80 femtomoles of HBP. Comparative derivatization of HPB was performed using O-bis(Trimethylsilyl)-trifluoroacetamide (BSTFA) and 2, 3, 4, 5, 6-Pentafluorobenzoylchloride (PFBC). Both Negative CI and Positive CI modes of analysis were compared to the more widely accepted EI modes of mass spectrometric analysis.

## Introduction

Smoking during pregnancy has been linked to a variety of adverse pregnancy outcomes, including low birthweight, spontaneous abortion, and infant death (Bajanowski, Brinkmann et al. 2007; Carter, Paterson et al. 2007; Jaddoe, de Ridder et al. 2007; Wilkinson and Walker, 2007). In addition, premature delivery (<36 weeks gestational age) has been linked to maternal smoking during pregnancy (Kolas, Nakling et al. 2000; Williams, Davenport et al. 2000; Raatikainen, Huurinainen et al. 2007). Some biological mechanisms which have been clinically confirmed link cigarette smoke to fetal health and include an association between nicotine and decreased placental blood flow and an increase in fetal heart rate. Of the fetal outcomes documented, low birthweight shows the clearest and most consistent association with maternal smoking (Mathews, 2001; Wideroe, Vik et al. 2003; Dubois and Girard, 2006; Giglia, Binns et al. 2006; Maccabe, Martinsson et al. 2007; Ng and Zelikoff, 2007). Evidence suggests that a dose response relationship exists between cigarette consumption, especially during the third trimester and birth weight. The prevalence of smoking during pregnancy has been estimated at between 15 and 30% of all pregnant women with the percentages varying slightly dependent on the source of the data used and with State-to-State variations (Kentucky generally ranks among the highest States in % of women smokers) (Pauly, Sparks et al. 2004; Bada, Das et al. 2005; Okoli, Kelly et al. 2007). Recently the Centers for Disease Control (CDC) reported that the incidence of State-specific smoking prevalence among U.S. adults varied widely ranging from a low of 14.2 percent in Utah to a high of 30.8 percent in Kentucky (Garcia-Rill, Buchanan et al. 2007; Mossey, Davies et al. 2007; Nigg and Breslau, 2007; Raatikainen, Huurinainen et al. 2007; Whitfield, King et al. 2007). In addition, women with mistimed or unwanted pregnancies are more likely to smoke throughout pregnancy as are women that are single and of teenage years. Smoking cigarettes during pregnancy has been shown to increase the risk of numerous adverse pregnancy outcomes, including low birthweight, preterm delivery, miscarriage, ectopic (tubal) pregnancy, infant death, low Apgar scores, and early childhood illness (respiratory illness, asthma) (Chen, Wen et al. 2007; Mahe, Girszyn et al. 2007; Ng and Zelikoff, 2007; Okoli, Kelly et al. 2007; Silver, 2007)

### The effects of tobacco exposure on neonatal growth and development

Prenatal tobacco exposure has been reported to be a significant risk factor for sudden infant death syndrome and is estimated to be responsible for up to 4,800 infant deaths as well as 61,000 low-birth-weight infants and 26,000 infants requiring neonatal intensive care annually (Yuan, Platt et al. 2005; Shah, Sullivan et al. 2006; Bajanowski, Brinkmann et al. 2007; Bashiri, Lazer et al. 2007; Raatikainen, Huurinainen et al. 2007). In a national survey of pregnant adult women, however, 20.4 percent reported smoking cigarettes during pregnancy. This proportion rises to about one-half for women in lower socioeconomic populations. Smoking during pregnancy is more prevalent among Caucasian women compared with African-American or Hispanic women (Bibby and Stewart, 2004; Chang, Selvin et al. 2006; Bell, Zimmerman et al. 2007; Benhadi, Wiersinga et al. 2007; Collins, David et al. 2007; Honein, Rasmussen et al. 2007). Caucasian women also smoke at higher levels than do women of other ethnicities. Women who smoke during pregnancy are less likely to be married, have less education, have lower incomes, and attend fewer prenatal visits compared with women who do not smoke during pregnancy (Singh and Kogan, 2007).

In a recent study of pregnant teenagers, more than one-half of whom were smokers, prenatal tobacco exposure was significantly related to reduced birth weight, birth length, head circumference, and chest circumference (Dewan, Brabin et al. 2003; Cornelius, Leech et al. 2004; Jones, Williams et al. 2004; Strinic, Bukovic et al. 2005; Leiner, Villa et al. 2007). These reductions were even more pronounced than those found in a similar cohort of the children of adult women. For example, in the study of adult mothers and their children, prenatal tobacco use was significantly associated with a reduction in birth weight of 158 grams per pack per day. In the children of teenage mothers, prenatal tobacco exposure was significantly associated with a reduction in birth weight of 202 grams per pack per day.

### Carcinogens found in tobacco

Thousands of chemicals are known to exist in tobacco smoke. These compounds range from small molecules such as ethylene oxide, butadiene, and styrene, to larger molecules such as the polycyclic aromatic hydrocarbons, tobacco specific nitrosamines, and aromatic amines (Hecht, 2004; Murphy, Link et al. 2004; Benowitz, Jacob et al. 2005; Bernert, Jain et al. 2005; Sturla, Scott et al. 2005; Carmella, Yoder et al. 2006; Hatsukami, Benowitz et al. 2006; Schwartz, Prysak et al. 2007). Cigarette smoking accounts for 30% of all cancer deaths in the United States with most of these deaths from lung cancer (Lippman and Spitz, 2001; Williams and Sandler, 2001; Fagerstrom, 2002; Hecht, 2003; Airoldi, Vineis et al. 2005). It has been established from numerous studies that cigarette smoke contains more than 40 known or putative carcinogens (Husgafvel-Pursiainen, 2004; Wogan, Hecht et al. 2004; Lemmonds, Hecht et al. 2005; Schwartz, Prysak et al. 2007). Many of these compounds have the potential because great harm in the developintg fetus and lead to unwanted effects after delivery.

N-nitrosamines (as shown in Table 1), are generated by the nitrosation of nicotine during tobacco curing and smoking are among the most abundant and potent carcinogens in tobacco smoke (Hoffmann, Rivenson et al. 1991; Hecht, Carmella et al. 1993; Hoffmann, Brunnemann et al. 1994; Brunnemann, Prokopczyk et al. 1996; Razani-Boroujerdi and Sopori, 2007). The tobacco specific N-nitrosamines NNN (N′-nitrosonornicotine) and NNK (4-(methylnitrosamino)-1-(3-pyridyl)-1-butanone) are particularly strong carcinogens in adult laboratory rodents, with a wide range of carcinogenic activity including a relatively high potency in the respiratory tract (Hoffmann, Brunnemann et al. 1994). The tobacco specific nitrosamine NNK is one of the most important carcinogens in tobacco smoke and smokeless tobacco products (LaVoie, Stern et al. 1987; Hoffmann, Rivenson et al. 1991; Brunnemann, Prokopczyk et al. 1996). This nitrosamine induces tumors of the lung, liver, nasal cavity, and pancreas in the rat. NNK is metabolically activated to reactive species that binds to hemoglobin and to DNA (Husgafvel-Pursiainen, 2002; Hecht, 2004).

### Carcinogenicity

N-Nitrosonornicotine is reasonably anticipated to be a human carcinogen based on sufficient evidence of carcinogenicity in experimental animals (Hecht, Carmella et al. 1993; Hoffmann, Brunnemann et al. 1994). N-nitrosonornicotine was shown to induce esophageal carcinomas and papillomas and carcinomas of the nasal cavity in rats of both sexes, nasal cavity adenocarcinomas in female rats, and papillomas of the nasal cavity and trachea in hamsters of both sexes (Hoffmann, Brunnemann et al. 1994) as well as epitheliomas and squamous cell carcinomas of the nasal cavity, and squamous cell carcinomas of the esophagus in male rats (Hoffmann, Rivenson et al. 1991; Hecht, Carmella et al. 1993). Intraperitoneal injection of N-nitrosonornicotine induced multiple pulmonary adenomas in mice of both sexes, lung adenomas in female mice, and nasal cavity tumors and tracheal papillomas in male hamsters (Hecht, 1997; Razani-Boroujerdi and Sopori, 2007).

### Exposure

N-Nitrosonornicotine has been found in a variety of tobacco products (chewing tobacco, snuff, cigarettes, and cigars), in mainstream and sidestream smoke from cigars and cigarettes, in saliva of chewers of betel quid with tobacco, and in saliva of oral-snuff users. Some of the N-nitrosonornicotine in saliva appears to be formed endogenously from nitrite in saliva and tobacco alkaloids. Thus, there is widespread exposure to N-nitrosonornicotine among users of tobacco products and those exposed to sidestream smoke. N-Nitrosonornicotine is reported to be produced by nitrosation of nicotine during the curing, ageing, processing, and smoking of tobacco. About half of the N-nitrosonornicotine originates in the unburnt tobacco, whereas the remainder is formed during burning. N-Nitrosonornicotine has been found in cigarettes at concentrations of 0.3 to 9 mg/kg, in snuff products at 12 to 29 mg/kg, in chewing tobacco at 3.5 to 90.6 mg/kg, and in cigarette smoke at 0.14 μg/cigarette (Preston-Martin, 1987; Hoffmann, Brunnemann et al. 1994; Brunnemann, Prokopczyk et al. 1996; Razani-Boroujerdi and Sopori, 2007)

### Biological markers of tobacco exposure

Numerous methods exist to monitor tobacco smoke in experimental animals as well as in human populations. The simplest method the following tobacco exposure is by assessing either urinary or serum cotinine levels. Although this provides an accurate assessment of short-term exposure to tobacco, it fails to provide a long term assessment of exposure over several weeks and/or months. Additional methods of exposure to tobacco include measurement of levels of specific tobacco smoke compounds in both the urine and/or serum. Again that this method is limited on the fact that it is a short term assessment of tobacco exposure and is highly dependent upon the metabolic elimination parameters for each compounds and in each individual. DNA adducts also provide effective means of monitoring exposure to tobacco smoke carcinogens. An ideal biomarker for use in assessing chemical exposures over a long-term basis is the use of the red cell protein hemoglobin. Hemoglobin not only serves his as a biomarker of exposure assessment, but also has a main function in the transport oxygen throughout the body into the various cells. Formation of electrophilic metabolites of carcinogens has been shown to react with a variety of nucleophilic sites on hemoglobin, therefore forming covalent adducts which persists for the life of the red cell (120 days) and thus can be detected and used as a biomarker of exposure. Hemoglobin adducts originally were suggested as a biochemical monitor of carcinogen exposure and a measure of genotoxic risk by Ehrenberg, Osterman-Golkar and others (Osterman-Golkar, Kautiainen et al. 1991; Ehrenberg and Tornqvist, 1992; Hecht, Carmella et al. 1993). Subsequentlly, a number of studies by several investigators. (Pastorelli, Guanci et al. 1999; Albertini, Sram et al. 2001; Jones, Liu et al. 2005; Beyerbach, Rothman et al. 2006; Ogawa, Oyama et al. 2006) have demonstrated that carcinogenic compounds, especially those present in cigarette smoke, bind covalently to hemoglobin. Hemoglobin presents itself as an ideal target for the interaction of various xenobiotics. Hemoglobin is relatively easy to obtain and the adducts of hemoglobin are stable adducts, unaffected by repair mechanisms that remove carcinogen adducts from DNA. Hemoglobin, in the red cell, has a lifetime in the circulation of approximately 120 days. Therefore, levels of hemoglobin adduct will accumulate in the circulation, thus the detection of the adduct and allowing the use of hemoglobin as a biomonitor of exposure to various agents, such as those carcinogens found in tobacco smoke.

Several reports have focused on the tobacco specific nitrosamine-DNA adducts in smokers and non-smokers in animal models using GC/MS and LC/MS-MS techniques (Lao et al. 2006; Guza et al. 2006; Thomson et al. 2004). However, very few studies have been carried out studies investigating the formation of these biomarkers in a variety of classifications of smoking populations, including passively smoke exposed individuals, as well as maternal-fetal pairs (Hecht et al. 1991; Carmella et al. 1990). The majority of the methods that have been previously applied for the characterization of nitrosamine hemoglobin adducts involved a multistep process of extraction and chromatographic isolations prior to analysis utilizing GC/MS (Hecht, Carmella et al. 1991; Falter, Kutzer et al. 1994; Atawodi, Lea et al. 1998). These methods for the most part were extremely time-consuming, and had limited practicality and clinical situations where hundreds of samples are being run simultaneously. The goal of our study was to develop a new sensitive method which would involve simple extraction techniques without a chromatographic clean up prior to GC/MS operation.

### Materials and methods

4-Hydroxy-1-(3-pyridyl)-1-butanone (HPB) and Deuterated (3, 3, 4, 4-D_4_)HPB were purchased from Toronto Research Chemicals Inc., (North York, ON, Canada), and O-bis(Trimethylsilyl)-trifluoroacetamide (BSTFA) was purchased from Pierce Biotechnology Inc., (Rockford, IL), 2, 3, 4, 5, 6-Pentafluorobenzoylchloride (PFBC) was purchased from Sigma-Aldrich Inc., (St Louis, MO). All additional chemical and solvents used throughout the experiments were of the highest grade commercially available.

### Patient selection

Patients were selected from those admitted to labor and delivery at Norton’s Suburban Hospital and Norton’s Downtown Hospital and. In 2004, Norton’s Hospitals delivered approximately 4,000 babies, placing it among the largest delivery centers in the metro Louisville area. The hospital’s population admitted for labor and delivery consists of approximately 30% smokers, ranging from less than one pack per day smokers to greater than two packs per day smokers. During initial admission of the mother, 10 mL of blood was obtained using heparinized vautainers for measurement of hemoglobin adduct concentrations. Following delivery of the infant, 10 mL of blood was obtained from the umbilical cord for measurement of hemoglobin adduct concentrations. All samples were acquired using a University of Louisville approved institutional review Board protocol.

### Maternal and fetal blood samples

Maternal blood samples were stratified according to smoking status (nonsmokers, 1 pk/day, and >1 pk/day). Samples were refrigerated immediately and stored until preparation of hemoglobin for analysis. Smoking status was assessed by detailed questionnaire as well as by measurement of plasma cotinine levels. Blood samples were centrifuged at 3,000 ×g for 10 minutes to generate packed red blood cells. Red cells were washed three times with 0.9% saline and lysed by the addition of 15 mL ice cold deionized water with vigorous shaking.

### Cotinine assessment

Cotinine levels were measured by immunoassay (BioQuant, San Diego, CA). Stratification of smokers into the smoking categories relied on cotinine assessments as well as questionnaire data. Non-smokers were judged as those women having cotinines less than 10 ng/mL, in accordance with previously published reports (Wagenknecht, Burke et al. 1992; McDonald, Perkins et al. 2005). Although widely used in epidemiological studies, self-report has been shown to underestimate the prevalence of cigarette smoking in some populations. In recent studies, self-report of cigarette smoking was validated against a biochemical marker of nicotine uptake, cotinine. The prevalence of smoking was slightly lower when defined by self-report (30.9%) than when defined by cotinine levels equal to or greater than 14 g/mL (32.2%, P < 0.05). The misclassification rate (proportion of reported nonsmokers with cotinine levels of at least 10 ng/mL) was 4.2% and was significantly higher among subjects who were African-American, had a high school education or less, or were reported former smokers. Possible reasons for misclassification include reporting error, environmental tobacco smoke, and an inappropriate cutoff point for delineation of smoking status. Using self-report as the gold standard, the cotinine cutoff points that maximized sensitivity and specificity were 14, 9, and 15 ng/mL for all, Caucasian, and African-American subjects, respectively. The misclassification rate remained significantly higher in African-American than in Caucasian subjects using these race-specific criteria. Misclassification of cigarette smoking by self-report was low in these young adults; however, within certain race/education groups, self-report may underestimate smoking prevalence by up to 4%.

### Analysis of tobacco specific nitrosamine maternal and fetal hemoglobin adducts

Hemoglobin solutions (maternal and fetal), 5–8 mls, corresponding to 4–6 mls blood were added to a 50 mL disposable borosilicate centrifuge tube. The normality of the solution was adjusted to 0.15 N with 1N NaOH. The solution was sonically dispersed for 1 hour at room temperature and the pH of the solution was adjusted to pH 6–7 by the addition of 1N HCl. A solution of [4, 4-D2]HPB (150 fmol) in 10 μl water was added as the internal standard. The mixture was centrifuged at 2000–3000 rpm for 10 minutes at room temperature and the supernatant was transferred with a Pasteur pipette to a second 50 mL centrifuge tube. The pH of the solution was adjusted to 1.5–2.5 by the addition of 1 N HCl and the solution extracted twice with equal volumes of dichloromethane and twice with equal volumes of hexane After discarding your organic extracts, the pH of the aqueous portion was adjusted to pH 6–7 with 1N NaOH, and was extracted three times with equal volumes of dichloromethane. The combined dichloromethane extracts were evaporated under reduced pressure and the residue was re-dissolved in 1 mL methanol, transferred to a small sample vial, and the methanol evaporated under nitrogen.

### Sample preparation HPB-hemoglobin adduct assay by GC/MS

100 μL lysed hemoglobin solutions were placed in a 7 mL borosilicate glass tube with screw cap telflon liner. 750 μL HPLC grade water and 150 μL 1 N NaOH were added to the hemoglobin solution. 10μL of 2.5 μg/mL (3, 3, 4, 4-D_4_)HPB was added as the internal standard (IS). The solution was incubated in air at 50 °C for 2 hours. Following incubation, the hemoglobin solution was acidified by addition of 225 μL 1 N HCl, and washed by the addition of 2 mL dichloromethane and 2 mL n-hexane. The washed solution were neutralized by addition of 75 μL 1 N NaOH. After washing, the samples were extracted twice with 2 mL dichloromethane, the extracts combined and dried under a gentle stream of nitrogen gas, and stored at −20 °C.

Stock solutions of HPB and (3, 3, 4, 4-D_4_)HPB were prepared in acetonitrile at concentrations of 1.25, 5.0, 12.5, 50.0, 125.0 and 500.0 ng/mL. Trimethylsilyl derivatization of HPB and (3, 3, 4, 4-D_4_)HPB were prepared by the addition of 10 μL BSTFA and 30 μL acetonitrile to samples followed by incubation in air for 1 hour at 60 °C. HPB-pentafluorobenzoate derivatization was performed by addition of 1 μL PFBC in 49 μL n-hexane:triethylamine (50:1) followed by a 1 hour incubation in air at 60 °C. The derivatized samples were transferred to gas chromatographic and mass spectrometric sample vials and were diluted to a total volume of 100 μL with acetonitrile. Samples were analyzed in triplicate using both negative and positive chemical ionization modes as well as electron impact ionization modes and reported as pmoles HPB/gm Hb ± SEM.

### GC/MS methods

A HP 6890 GC coupled to HP 5973 MS was interfaced with the Hewlett-Packard ChemStation software package for data acquisition and analysis. GC conditions were optimized with a temperature programming to attain the highest sensitivity and resolution. Helium carrier gas was used with a flow rate 1.3 mL/min. Gas chromatographic conditions included injection of samples using splitless injection mode with an injection port temperature at 280 °C. A GC capillary column consisting of a DB-15MS with dimensions 15 m × 0.25 mm id × 0.25 μm film thickness (J&W Scientific, Folsom, CA). Samples were eluted through the GC column using a temperature program consisting of an initial temperature of 50 °C for one minute, followed by a ramp increase in temperature to 230 °C at 20 °C/min, followed by a final isothermal temperature at 230 °C for five minutes.

### Positive ion chemical ionization (PICI)

Full scan (m/z 50–450) PICI mass spectral data (Table 2) were acquired to characterize derivatized HPB and (3, 3, 4, 4-D_4_)HPB. (3, 3, 4, 4-D_4_) HPB was used as internal standard (IS). Mass spectrometer conditions consisted of electron energy 221 eV, source temperature 250 °C, emission current 237 μamp, and electron multiplier voltage 1718 V. Quantitation of trimethylsilyl derivative of HPB by BSTFA was performed using selected ion monitoring (SIM) at m/z 238 and 242 for analyte and IS respectively. Pentafluorobenzoate derivatized HPB by PFBC was also characterized using SIM at m/z 360 and 364 for analyte and IS respectively.

### Negative ion chemical ionization (NICI)

Full scan (m/z 50–450) NICI mass spectral data (Table 3) were acquired to identify derivatized HPB and (3, 3, 4, 4-D_4_)HPB. Mass spectrometer conditions consisted of electron energy 193 eV, source temperature 150 °C, emission current 49 μamp, and electron multiplier voltage 2996 V. Quantitation of the trimethylsilyl derivative of HPB was performed using selec ted ion monitoring (SIM) at m/z 237 and 247 for analyte and IS respectively. The HPB-pentafluorobenzoate derivative was also characterized using SIM at m/z 359 and 363 for analyte and IS respectively.

### Electron impact ionization (EI)

Full spectrum (m/z 50–450) electron impact ionization (EI) mass spectral data (Table 4) were acquired to characterize derivatized HPB and (3, 3, 4, 4-D_4_)HPB. Electron impact ionization conditions included electron energy 70 eV, source temperature 230 °C, emission current 35 μamp, and electron multiplier voltage 1859 V. Quantification of derivatized HPB by BSTFA was performed using selected ions monitoring (SIM) at m/z 222 and 226 for analyte and IS respectively.

## Results

Positive ion chemical ionization (PICI), negative ion chemical ionization (NICI) and electron impact ionization (EI) techniques were investigated to develop a sensitive gas chromatographic/mass spectrometry (GC/MS) procedure for the assessment of HPB (4-Hydroxy-1-(3-pyridyl)-1-butanone) released from hemoglobin adducts upon base catalyzed hydrolysis. Derivatization techniques using BSTFA (N,O-bis(Trimethylsilyl)-trifluoroacetamide) and PFBC (2, 3, 4, 5, 6-Pentafluorobenzoylchloride) were also compared to improve the detection of HPB.

**Figure 1:**
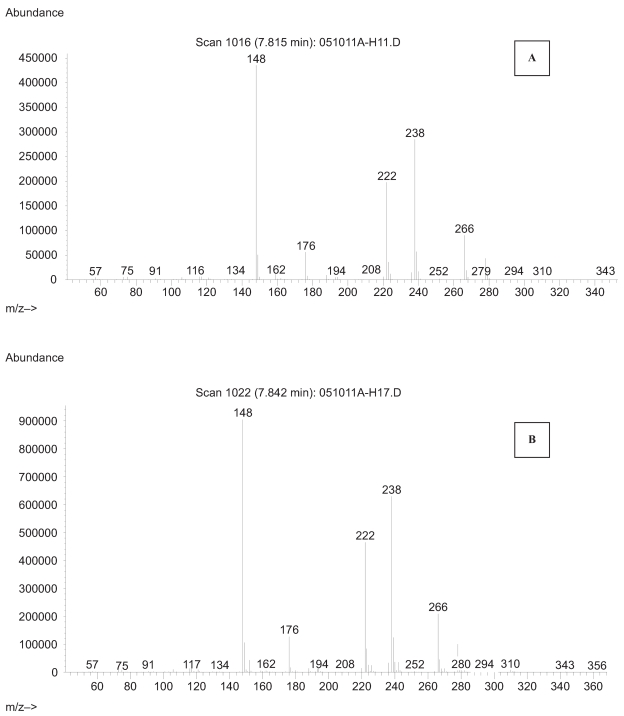
Positive ion chemical ionization spectra of trimethylsilyl derivatized 4-hydroxy-1-(3-pyridyl)-1-butanone (HPB) isolated from maternal smokers blood (>1 pack/day) (**A**) compared to the spectra of authentic derivatized HPB shown in **B**.

PICI is a soft ionization technique which generates a strong parent molecular ion along with relatively few fragmentation ions. In PICI, the reagent gas is ionized by collision with emitted electrons from the electron source. The reagent gas ions then react chemically with the gas molecules obtained from the sample during volatilization to form positively charged molecular ion containing an extra proton [M+H]^+^. A full scan m/z 50–450 PICI mass spectra for derivatized HPB by BSTFA was obtained by GC/MS and is illustrated in Figure 1, illustrating the molecular ions [M+H]^+^ of 238 and m/z 222 which corresponds to [M-CH_3_]^+^ whereas m/z 148 corresponds to the [M-OSi(CH_3_)_3_]^+^ product.

**Figure 2:**
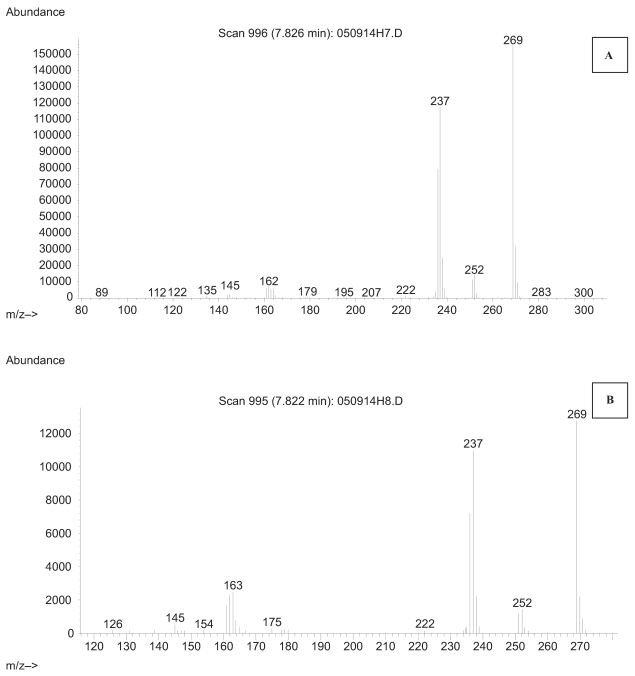
Negative ion chemical ionization spectra of trimethylsilyl derivatized 4-hydroxy-1-(3-pyridyl)-1-butanone (HPB) isolated from maternal smokers blood (>1 pack/day) (**A**) compared to the spectra of authentic derivatized HPB shown in **B**.

Negative ion chemical ionization (NICI) is a relatively soft ionization technique which produces parent molecular ions with relatively limited fragmentation of the parent molecule. As was similar with positive ion chemical ionization, negative ion chemical ionization produces low energy thermal electrons. Sample molecules absorb these thermal electrons and thus form negatively charged molecular ions M^−^. NICI is more sensitive than PICI because of lack of formation of negative reagent gas ions. A full scan m/z 50–450 NICI mass spectra for derivatized HPB by BSTFA was obtained by GC/MS is shown in Figure 2, illustrating the parent molecular ion of m/z 237 with limited fragmentary ions.

**Figure 3:**
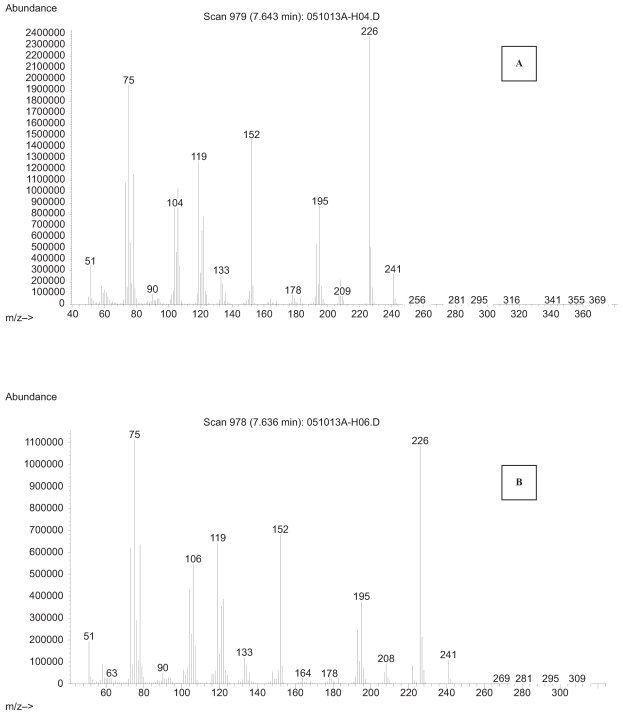
Electron impact ionization spectra of trimethylsilyl derivatized 4-hydroxy-1-(3-pyridyl)-1-butanone (HPB) isolated from maternal smokers blood (>1 pack/day) (**A**) compared to the spectra of authentic derivatized HPB shown in **B**.

Electron impact ionization (EI) is a hard ionization technique compared to either positive or negative chemical ionization modes and this technique generates relatively small amounts of parent molecular ions but considerably larger quantities of molecular fragmentary ions. In this technique, the generation of both parent molecular ions and considerable fragmentary and patterns can be used to characterize not only chemical nature of the given unknown but also to some degree structural assignments. A full scan m/z 50–450 NICI mass spectra for derivatized HPB by BSTFA was obtained by GC/MS illustrating a molecular ion of molecular ion M^+.^ m/z of 237 and fragmentary ions of a base peak of m/z 222 [M-CH_3_] is shown in Figure 3.

Similar values of both maternal and fetal nitrosamine adducts were detected both and African-American as well as Caucasian populations. The relationship between the formation of nitrosamine maternal adducts as well as fetal nitrosamine adducts were found to the similar both ethnic diversities. Although the placenta is sometimes considered a barrier that protects the infant from exposures to exogenous chemicals and substances, it is clear that the placental linings are not effective in blocking the transfer of hazardous chemicals, including the chemical carcinogens found in tobacco, from the fetus. Approximately 50% of maternal exposure to the tobacco specific nitrosamines was found to cross the placenta and he found adducted to fetal hemoglobin. Since there is no maternal or fetal transfer of blood this tends to indicate that either the parent nitrosamine has crossed into the fetal circulation via the placenta and become metabolized to reactive intermediates in the fetal tissue or a reactive metabolite of the nitrosamines has been formed in the maternal component and crossed into the fetal circulation. Considering the reactivity of various selector files of chemical carcinogens, including nitrosamines, it is highly probable that the parent nitrosamine has crossed from the maternal component into the fetal component. These studies clearly demonstrate that fetal exposure to environmental carcinogens can occur as a result of maternal exposures during pregnancy. Although the samples were acquired at delivery, the study suggests that mothers and smoke during pregnancy are potentially exposing their unborn fetuses to carcinogenic as well as toxic chemicals even during the early stages of pregnancy as a result of crossing of these carcinogens through the placenta. Future studies will be directed at determining the relationships between maternal and fetal exposure to environmental as well as tobacco-related carcinogens particularly in the early stages of pregnancy, particularly the first trimester, where a greater activity of cell division, and organogenesis is occurring. In addition, feature studies will be directed at determining the relationship between both maternal and fetal exposure to tobacco related carcinogens of a variety of classes and a variety of ethnicities. These studies will include the determination of hemoglobin adducts in both maternal and fetal circulations, as well as the differentiation between phase 1 and phase 2 enzyme levels in both maternal and fetal tissues that are related to the vile activation as well as they to detoxification of a variety of chemical carcinogens. Taken together these studies will add considerably to the information available concerning hazardous effects of chemical carcinogens, including tobacco-related carcinogens, on pregnancy and neonatal development.

## Discussion

A comparative study for PICI, NICI and EI was performed to evaluate the suitability of GC/MS method for the detection of hemoglobin adducts to tobacco specific nitrosamines in human samples. The results indicate that both negative ion chemical ionization as well as positive ion chemical ionization modes of detection and mass spectrometry provided greater sensitivity and detection of the released HBP from hemoglobin in both maternal and fetal samples. Both ionization techniques produce considerably less background noise as compared to electron impact mode of ionization and the signal-to-noise ratio in both maternal and fetal samples exceeded 20:1 in the chemical ionization modes compared to 3:1 in the electron impact ionization modes. Although both negative ionization as well as a positive ionization modes appear to be considerably more accurate and sensitive than the electron impact mode of ionization for the analysis of nitrosamine adducts of hemoglobin, the electron impact mode of ionization does afford benefits when compared to chemical ionization modes. Analysis of samples using electron impact mode of ionization allows the investigator to obtain not only parent molecular ions of both derivatized and non-derivatized samples, but also allows the obtaining of multiple fragmentary ions which can be used in structure elucidation and characterization of unknown adducts.

Both HPB and (3,3,4,4-D_4_) HPB were derivatized by BSTFA and PFBC separately to improve the sensitivity of the compounds. These agents are well-established chemicals used for enhancing detection of chemicals using gas chromatographic and mass spectral techniques and improve the detection sensitivity over the non-derivatized sample by approximately tenfold. In addition, the process of derivatization of the released HBP forms a highly volatile chemical that is easier to process and analyzed using the described gas chromatographic techniques. Both derivatization techniques utilizing both BSTF at a as well as PFBC enhanced selectivity and sensitivity of detection when compared to non-derivatized samples. BSTFA derivatives are characterized as trimethylsilyl products of the hydroxyl group of HPB. On the other hand, derivatives performed with PFBC are characterized as a pentafluorobenzoate derivative of HPB. Comparative studies between the two derivatization techniques and among all three ionization techniques showed that the PFBC derivatives of HBP resulted in higher degree of sensitivity in the detection of both maternal and fetal nitrosamine hemoglobin adducts.

This study demonstrates that upon a mild base hydrolysis HPB was released from lysed hemoglobin obtained from smoking and non-smoking mothers and fetal cord blood. In addition, increasing levels of both maternal and fetal tobacco specific nitrosamines, as assessed via the detection of HBP released upon basic hydrolysis of hemoglobin, was found as smoking status increased from non-smokers to greater than one pack per day smokers. These studies indicate that mothers and smoke during pregnancy exposed the unborn fetus to compounds found in tobacco, including the tobacco specific nitrosamines, many of which have been shown to cause animal tumors and teratogenesis. This study strongly confirms that the placenta does allow the passing of carcinogens, such as those found in tobacco, into the fetus. While the study was limited in the obtaining of maternal and fetal samples at term, this does not preclude that damage can in fact occurred to the developing fetus as a result of maternal smoking habits during pregnancy.

Previous studies determining smoking status in both individuals as well as during pregnancy has been limited in the past to determining either urinary or serum levels of a specific metabolite of nicotine found in biological fluids, namely cotinine. Numerous assays and reports have been published over the many years dealing with this biomarker of tobacco exposure and in large parts this is been an effective biological indicator of smoke exposure. However there are many limitations as to the applicability and use of cotinine as a biomarker of tobacco exposure. One of the downsides of using cotinine as a specific biomarker of tobacco exposure is that the half-life of cotinine is relatively short in biological systems, approximately 24 hours. The use of cotinine alone as a biomarker of tobacco exposure can lead to conflicting results when one takes into consideration the potential of both long and protracted labor in childbirth. A more recent biological marker, such as that of using protein adducts of tobacco specific compounds provide a much clearer and much more precise assessment of current smoking status that is not interfered upon by use of half-life of cotinine. These results illustrate the applicability and use of hemoglobin as a biomarker of tobacco specific nitrosamines found in tobacco smoke. In addition, we have shown that the methods that are used allow both sensitive and accurate assessments of this biomarker in both maternal as well as in fetal blood samples taken at delivery. By assessing not only the maternal exposure during pregnancy but also fetal exposures taken at the time of delivery we may be in better position to assess the potential harm of these compounds during fetal gestation and delivery.

Future studies will be directed at determining the efficacy of various modes of detection for environmental carcinogen adducts on hemoglobin, especially tobacco-related carcinogens, and the overall effect that these compounds have in the developing fetus. Specifically, we will be investigating early first trimester exposure utilizing various biological markers such as hemoglobin adducts and amniotic fluid samples as indicators of exposure to harmful chemicals early in pregnancy. By understanding the relationship between exposure assessment, biological markers, and risk of potential disease, we may be in a better position to both predict and prevent the potential carcinogenic effects associated with maternal smoking during pregnancy.

## Figures and Tables

**Table 1:** Tobacco specific nitrosamines and their carcinogenic rating according to the International Agency for Research on Cancer (Griciute, 1976; O’Neill and Fishbein, 1986; Preston-Martin, 1987).

Nitrosamine	Abbreviation	Carcinogenicity
N-nitrosonornicotine	NNN	Group 2B IARC
4-(methylnitrosamino)-1-(3-pyridyl)-1-butanone	NNK	Group 2B IARC
4-(methylnitrosamino)-1-(3-pyridyl)-1-butanol	NNAL	Group 2B IARC
N′-nitrosoanatabine	NAT	Group 3 IARC
N′-nitrosoanabasine	NAB	Group 3 IARC
4-(methylnitrosamino)-4-(3-pyridyl)butanal	NNA	Group 3 IARC
4-(methylnitrosamino)-4-(3-pyridyl)-1-butanol	iso-NNAL	
4-(methylnitrosamino)-4-(3-pyridyl)butyric acid	iso-NNAC	

**Table 2:** Quantification of HPB derivatized with BSTFA in maternal and Fetal cord blood samples using PICI mass spectrometry (mean ± SEM).

Smoking Status	Maternal (pmols HPB/gm Hb)	Maternal (pmols HPB/gm Hb)	Fetal (pmols HPB/gm Hb)	Fetal (pmols HPB/gm Hb)
Non-Smokers (n = 45)	0.52 ± 0.22	0.42 ± 0.13	0.25 ± 0.13	0.13 ± 0.06
Passively Exposed (n = 26)	2.16 ± 0.98	1.86 ± 0.35	1.31 ± 0.75	0.64 ± 0.35
<1 pack per day smokers (n = 83)	5.24 ± 1.35	4.77 ± 0.68	2.16 ± 0.86	1.52 ± 0.74
>1 pack per day smokers (n = 91)	12.65 ± 2.41	16.32 ± 1.16	6.52 ± 1.63	7.86 ± 1.55

**Table 3:** Quantification of HPB derivatized with BSTFA in maternal and Fetal cord blood samples using NICI mass spectrometry (mean ± SEM).

Smoking Status	Maternal (pmols HPB/gm Hb)	Maternal (pmols HPB/gm Hb)	Fetal (pmols HPB/gm Hb)	Fetal (pmols HPB/gm Hb)
Non-Smokers (n = 45)	0.74 ± 0.36	0.63 ± 0.55	0.41 ± 0.25	0.36 ± 0.29
Passively Exposed (n = 26)	2.88 ± 0.92	2.37 ± 1.53	1.55 ± 0.72	1.24 ± 0.85
<1 pack per day smokers (n = 83)	6.44 ± 2.43	5.72 ± 1.92	3.11 ± 1.44	2.79 ± 1.29
>1 pack per day smokers (n = 91)	15.23 ± 4.83	15.33 ± 6.22	5.16 ± 3.26	5.13 ± 2.78

**Table 4:** Quantification of HPB derivatized with BSTFA in maternal and Fetal cord blood samples using EI mass spectrometry (mean ± SEM).

Smoking Status	Maternal (pmols HPB/gm Hb)	Maternal (pmols HPB/gm Hb)	Fetal (pmols HPB/gm Hb)	Fetal (pmols HPB/gm Hb)
Non-Smokers (n = 45)	n.d.	n.d.	n.d.	n.d.
Passively Exposed (n = 26)	1.66 ± 2.33	2.15 ± 2.04	1.86 ± 1.32	1.89 ± 1.42
<1 pack per day smokers (n = 83)	9.22 ± 6.24	7.29 ± 3.71	4.64 ± 2.88	3.94 ± 2.36
>1 pack per day smokers (n = 91)	19.4 ± 12.36	17.3 ± 9.34	11.6 ± 7.82	12.7 ± 10.4
